# Dating the Origin and Estimating the Transmission Rates of the Major HIV-1 Clusters in Greece: Evidence about the Earliest Subtype A1 Epidemic in Europe

**DOI:** 10.3390/v14010101

**Published:** 2022-01-06

**Authors:** Stefanos Limnaios, Evangelia Georgia Kostaki, Georgios Adamis, Myrto Astriti, Maria Chini, Nikos Mangafas, Marios Lazanas, Stavros Patrinos, Simeon Metallidis, Olga Tsachouridou, Vasileios Papastamopoulos, Eleni Kakalou, Dimitrios Chatzidimitriou, Anastasia Antoniadou, Antonios Papadopoulos, Mina Psichogiou, Dimitrios Basoulis, Maria Gova, Dimitrios Pilalas, Dimitra Paraskeva, Georgios Chrysos, Vasileios Paparizos, Sofia Kourkounti, Helen Sambatakou, Vasileios Bolanos, Nikolaos V. Sipsas, Malvina Lada, Emmanouil Barbounakis, Evrikleia Kantzilaki, Periklis Panagopoulos, Efstratios Maltezos, Stelios Drimis, Vana Sypsa, Pagona Lagiou, Gkikas Magiorkinis, Angelos Hatzakis, Lemonia Skoura, Dimitrios Paraskevis

**Affiliations:** 1 Department of Hygiene, Epidemiology and Medical Statistics, Medical School, National and Kapodistrian University of Athens, 11527 Athens, Greece; stalimn@hotmail.com (S.L.); ekostakh@med.uoa.gr (E.G.K.); marygova@gmail.com (M.G.); vsipsa@med.uoa.gr (V.S.); pdlagiou@med.uoa.gr (P.L.); gmagi@med.uoa.gr (G.M.); ahatzak@med.uoa.gr (A.H.); 2 1st Department of Internal Medicine, G. Gennimatas General Hospital, 11527 Athens, Greece; geo.adamis@gmail.com (G.A.); myrto-astriti@hotmail.com (M.A.); 3 3rd Department of Internal Medicine-Infectious Diseases Unit, “Korgialeneio-Benakeio” Red Cross General Hospital, 11526 Athens, Greece; mariachini@gmail.com (M.C.); nikosmangafas@gmail.com (N.M.); mklazanas@ath.forthnet.gr (M.L.); 4 National Public Health Organization, 15123 Marousi, Greece; stavrosspatrinos@gmail.com; 5 1st Department of Internal Medicine, AHEPA University Hospital, Medical School, Aristotle University of Thessaloniki, 54636 Thessaloniki, Greece; metallidissimeon@yahoo.gr (S.M.); olgat_med@hotmail.com (O.T.); 6 5th Department of Internal Medicine and Infectious Diseases, Evaggelismos General Hospital, 10676 Athens, Greece; vasileios.papastamopoulos@gmail.com (V.P.); ekakalou@yahoo.gr (E.K.); 7 National AIDS Reference Centre of Northern Greece, Department of Microbiology, Medical School, Aristotle University of Thessaloniki, 54124 Thessaloniki, Greece; dihi@med.auth.gr (D.C.); mollyskoura@gmail.com (L.S.); 8 4th Department of Medicine, Attikon University General Hospital, Medical School, National and Kapodistrian University of Athens, 12462 Athens, Greece; ananto@med.uoa.gr (A.A.); antpapa1@otenet.gr (A.P.); 9 1st Department of Medicine, Laikon General Hospital, Medical School, National and Kapodistrian University of Athens, 11527 Athens, Greece; mpsichog@yahoo.gr (M.P.); dimitris.bassoulis@gmail.com (D.B.); 10 Medical School, Aristotle University of Thessaloniki, 54124 Thessaloniki, Greece; pilalas_jim@hotmail.com; 11 Department of Internal Medicine, Tzaneio General Hospital, 18536 Piraeus, Greece; dimparaskeva@gmail.com (D.P.); gchrysos@gmail.com (G.C.); steliosdrimis@gmail.com (S.D.); 12 HIV/AIDS Unit, A. Syngros Hospital of Dermatology and Venereology, 16121 Athens, Greece; vpaparizos@yahoo.gr (V.P.); kourkounti@in.gr (S.K.); 13 HIV Unit, 2nd Department of Internal Medicine, Hippokration General Hospital, Medical School, National and Kapodistrian University of Athens, 11527 Athens, Greece; helensambatakou@msn.com (H.S.); billy_bolanos@hotmail.com (V.B.); 14 Department of Pathophysiology, Laikon General Hospital, Medical School, National and Kapodistrian University of Athens, 11527 Athens, Greece; nsipsas@med.uoa.gr; 15 2nd Department of Internal Medicine, Sismanogleion General Hospital, 15126 Marousi, Greece; malvinalada@gmail.com; 16 Department of Internal Medicine, University Hospital of Heraklion “PAGNI”, Medical School, University of Crete, 71110 Heraklion, Greece; barbuman2003@yahoo.gr (E.B.); eyrykleia3@yahoo.gr (E.K.); 17 Department of Internal Medicine, University General Hospital, Democritus University of Thrace, 68100 Alexandroupolis, Greece; ppanago@med.duth.gr (P.P.); emaltez@med.duth.gr (E.M.)

**Keywords:** human immunodeficiency virus (HIV), molecular epidemiology, phylodynamic analysis, date of origin, transmission rate, transmission clusters, Greece

## Abstract

Our aim was to estimate the date of the origin and the transmission rates of the major local clusters of subtypes A1 and B in Greece. Phylodynamic analyses were conducted in 14 subtype A1 and 31 subtype B clusters. The earliest dates of origin for subtypes A1 and B were in 1982.6 and in 1985.5, respectively. The transmission rate for the subtype A1 clusters ranged between 7.54 and 39.61 infections/100 person years (IQR: 9.39, 15.88), and for subtype B clusters between 4.42 and 36.44 infections/100 person years (IQR: 7.38, 15.04). Statistical analysis revealed that the average difference in the transmission rate between the PWID and the MSM clusters was 6.73 (95% CI: 0.86 to 12.60; *p* = 0.026). Our study provides evidence that the date of introduction of subtype A1 in Greece was the earliest in Europe. Transmission rates were significantly higher for PWID than MSM clusters due to the conditions that gave rise to an extensive PWID HIV-1 outbreak ten years ago in Athens, Greece. Transmission rate can be considered as a valuable measure for public health since it provides a proxy of the rate of epidemic growth within a cluster and, therefore, it can be useful for targeted HIV prevention programs.

## 1. Introduction

Although in 2021 we are in the midst of the SARS-CoV-2 pandemic, other contagious diseases such as HIV continue to be major global issues for which there is no cure or a vaccine. According to WHO estimates, 37.7 million have been living with HIV until the end of 2020, and 680,000 people died in the previous years due to HIV-related conditions (http://www.UNAIDS.org, accessed on 15 September 2021). In the absence of a vaccine, effective HIV prevention, early diagnosis and access to antiretroviral (ARV) treatment provide the only way to prevent HIV infections. One of the key actions for HIV prevention is to understand the characteristics of local epidemics (http://www.cdc.gov, accessed on 15 September 2021). In addition to traditional methods, molecular epidemiology has provided a new tool for a better understanding of the HIV-epidemic, and it is considered as one of the key actions for responding to emerging HIV clusters [1,2,3,4,5,6,7,8,9,10,11,12,13,14,15,16,17,18].

HIV-1 has been classified into different clades consisting of 9 pure subtypes A–D, F–H, J and K, which are further divided into sub-subtypes and intersubtype recombinant forms [19]. Traditionally, subtyping has been used to monitor viral genetic diversity and to map the distribution of HIV-1 clades across different geographic areas. Sophisticated phylogenetic and phylodynamic analyses have been implemented to investigate the characteristics of HIV dispersal (identification of clusters) and to estimate the clusters’ transmission dynamics over time [1,2,3,4,5,6,7,8,9,10,11,12,13,14,15,16,17,20,21,22].

The HIV-1 epidemic in Greece has been characterized by a rapid increase in the number of diagnoses among people who inject drugs (PWID) after 2010 [20,21,23]. The outbreak peaked in 2012 and declined thereafter reaching pre-outbreak diagnoses rates in 2016. Before 2011, the dominant risk group was men who have sex with men (MSM) (https://eody.gov.gr/, accessed on 15 September 2021). Although HIV-1 cases declined after 2012, the proportion of diagnoses among PWID remained higher (i.e., between 16.0% and 17.9%) than before the outbreak (around 5%) (https://eody.gov.gr/, accessed on 15 September 2021). Our previous analysis, using a dense sample of sequences from HIV-1 individuals sampled during 1999–2015, showed that the subtypes A1 and B were the most prevalent clades in Greece [22]. Regarding the patterns of HIV-1 transmission, 93.8% and 77.0% of sequences for subtype A1 and B, respectively, were found to belong within local clusters [22]. The date of the origin and the transmission rates of these clusters remained unknown. This information would be important to identify rapidly expanding clusters and, therefore, to provide a framework for implementing molecular analysis for HIV prevention.

Taking all the above into consideration, our aim was to estimate the date of the origin and the transmission rates of the major local clusters of subtypes A1 and B in Greece, using a nationwide dense sample collected during 1999–2015.

## 2. Materials and Methods

### 2.1. Study Population

The study population included all people living with HIV (PLHIV) (*n* = 6166) for whom protease (PR) and partial reverse transcriptase (RT) sequences were available during 1999–2015 [22]. This number (i.e., 6166 PLHIV) corresponds to 57.2% of all PLHIV diagnosed during the same time period in Greece [22]. In more detail, our study sample consisted of 4790 (77.7%) sequences sampled from central/southern Greece, 1298 (21.0%) sampled from northern Greece and 78 (1.3%) sampled from the island of Crete, as reported previously [22]. Phylodynamic analysis was performed only for sequences belonging to molecular transmission clusters (MTCs) of subtypes A1 and B, which are the prevalent clades in Greece. Briefly, MTCs were identified by phylogenetic analysis using a large set of globally sampled sequences as references, and their definition was based on the following two different criteria: (i) phylogenetic clusters including at least 2 sequences from Greece at a proportion higher than 70% of the total number of sequences within the cluster (tentative MTCs) (geographic criterion), and ii) large tentative MTCs consisting of more than 10 sequences receiving transfer bootstrap expectation (TBE) higher than 75% or posterior probability support higher than 0.85 (phylogenetic robustness criterion) [22]. Among the sequences classified within subtypes A1 (*n* = 1751) and B (*n* = 2575), 93.8% (1642 of 1751) of A1 sequences and 77.0% (1982 of 2575) of B sequences were found to belong to MTCs [22]. Phylodynamic analysis was performed in 1533 and 1560 sequences belonging to large MTCs including more than 10 sequences of subtypes A1 and B, respectively. Specifically, phylodynamic analysis was conducted on 2 subtype A1 and 31 subtype B MTCs. For subtype A1, the largest MTC of 1518 sequences was divided into 13 smaller clusters (subclusters) for the purpose of the analysis. The selection of the smaller clusters was performed according to their phylogenetic clustering in subclusters.

The study was approved by the Ethics committee of the Medical School of the National and Kapodistrian University of Athens (1516010195-08/12/2015).

### 2.2. Phylodynamic Analysis

For all the alignments, the final size was 897 nucleotides. The informative sites were calculated for a number of MTCs using the online tool DIVEIN (https://indra.mullins.microbiol.washington.edu/DIVEIN/index.html, accessed on 5 December 2021). In more detail, the informative sites (gaps excluded) for the subtype A1 MTCs were 13 for cluster 1 and 225 for cluster 13. For subtype B MTCs, the informative sites were 158 for cluster 10, 372 for cluster 30 and 14 for cluster 8. In the context of our phylodynamic analysis, all the MTCs (for both subtypes) were analyzed separately. The 31 MTCs of subtype B were independent clusters. Contrarily, for subtype A1, the 13 of 14 MTCs were parts of a larger cluster consisting of 1518 sequences [22].

Molecular clock calculation with phylodynamic analyses was applied on PR and partial RT sequences generated by Sanger sequencing and implemented in the BEAST program (version 1.8.0) [24,25]. The ambiguous positions were treated by BEAST as missing positions, and indels were removed from the alignments. We used the TempEst v1.5.1 program (http://tree.bio.ed.ac.uk/software/tempest/, accessed on 5 December 2021) to plot the root-to-tip genetic distance against sampling time for a number of MTCs with different proportions of ARV-treated PLHIV to verify that there was significant molecular clock signal.

Analyses were performed by using different nucleotide substitution models (GTR+G, HKY+G), uncorrelated lognormal relaxed clock models and the birth–death models [24,25], while non-informative priors were used for the Markov chain Monte Carlo (MCMC) runs. We performed ModelTest analysis for several alignments and the best-fitting models selected were the GTR+G or the TVM+R. Phylodynamic analysis was performed using the GTR+G which was the model selected by ModelTest and the HKY+G for the MTCs that GTR+G did not converge. In more detail, the MCMC analysis was run for each MTC for 80 × 10^6^ generations, sampled every 8000 steps with the first 10% of samples discarded as burn-in. MCMC convergence and effective samples sizes (ESS > 100) were checked by using the program Tracer v1.7.1 (http://tree.bio.ed.ac.uk/software/tracer/, accessed on 15 September 2021). To examine if the estimated highest posterior density (HPD) intervals would be narrower after merging different MCMC runs, we performed three independent runs by setting the length of chain at 80 × 10^6^ generations for each one of them for the subtype B cluster 29. Then, we used LogCombiner v2.1.3 program to combine the tree files from the three independent runs of BEAST. The pattern of branching was identical between the tree obtained by combining the tree files from the three independent runs and the tree obtained from the first run of our analysis. Regarding the estimated time to the most recent common ancestor (tMRCA) at the nodes of the two trees, they were almost identical [i.e., tMRCA (95% HPD) at the root of the tree obtained from the first run: 10.65 (8.55, 13.47); tMRCA (95% HPD) at the root of the tree obtained by the combination of the tree files from the independent runs: 10.62 (8.49, 13.44)].

The maximum clade credibility (MCC) tree was selected from the posterior tree distribution by the TreeAnnotator v2.1.3 program. The date of the origin of the MTC was approximated to the median estimate of the tMRCA of the root node of the dated tree. Molecular clock analysis was performed separately for each MTC and included all the available sequences within each one of them.

To investigate the effect of the inclusion of HIV sequences from ARV-treated PLHIV to molecular clock calculations, we examined the accuracy of dating in the 13 subclusters of the largest MTC of subtype A1. Specifically, 11 out of 13 subclusters consisted of sequences from ARV-treated PLHIV at proportions lower than 30%, and for the remaining 2 subclusters the corresponding proportions were 36% and 45%. For the two subclusters with the highest proportions of ARV-treated PLHIV, phylodynamic analysis was repeated after the merging of the subcluster of interest with another subcluster which was selected to be closely related to it according to the inferred phylogenetic tree. This merging resulted in a decrease in the proportion of ARV-treated individuals in both subclusters at levels lower than 30%.

Drug resistance sites were not removed since most sequences were sampled from drug naïve PLHIV. For MTCs with high proportion of ARV-treated PLHIV, we merged those MTCs with another MTC that resulted in a decrease in the proportion of ARV-treated individuals, and the molecular clock signal was found significant.

### 2.3. Estimation of Transmission Rates

Transmission rates were estimated only for ARV-treatment naïve PLHIV within each MTC, as described previously [26]. Specifically, transmission rate equals the number of transmission events (number of PLHIV in cluster—1) divided by the total HIV-infection person years within the cluster during which the observed transmissions occurred. The latter is calculated by the sum of all node ages plus the longest node age to account for the person-time of the founder person in the cluster [26]. The equation for transmission rate is described below:

$$\mathrm{Transmission}\;\mathrm{rate}=\frac{\left(\mathrm{number}\;\mathrm{of}\;\mathrm{people}\;\mathrm{in}\;\mathrm{cluster}\;-1\right)}{\sum \left(\mathrm{all}\;\mathrm{node}\;\mathrm{ages}\right)+\;\mathrm{longest}\;\mathrm{node}\;\mathrm{age}}$$

### 2.4. Statistical Analysis

Statistical analysis was performed by fitting a multivariable linear regression model on the transmission rate and other variables representing the characteristics of the MTCs. Specifically, the transmission rate was defined as the outcome variable on this model, while the number of sequences per cluster, gender (% of males), origin (% of Greeks), age (median estimate), region of sampling (% of sequences sampled in central/southern Greece), HIV-1 subtype (subtype A1, subtype B), risk group (MSM, PWID, non-MSM and non-PWID) and year of sampling (median estimate) were chosen as possible explanatory variables. In addition, the correlation between root-to-tip and sampling time was assessed by Pearson’s and Spearman’s correlation coefficients. The level of significance was set at 0.05. Analyses were performed in Stata 16.0-StataCorp LP software.

## 3. Results

### 3.1. Validation of Molecular Clock Calculations

**Figure 1:**
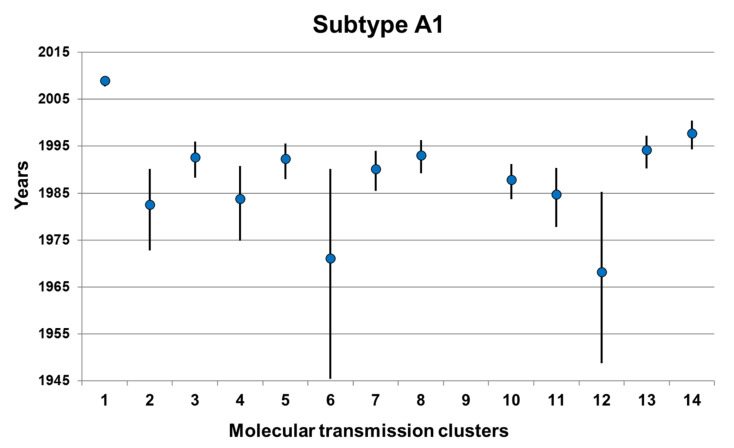
Molecular clock calculations of the time of the most recent common ancestor (tMRCA) for the 14 HIV-1 subtype A1 molecular transmission clusters (median, 95% HPD interval) where no cut-off was implemented for the proportion of the ARV-treated PLHIV within a cluster. Dots indicate the median estimations and horizontal lines the 95% HPD intervals.

Molecular clock calculations in the 13 subclusters of the largest A1 MTC with different proportions of ARV-treated individuals revealed that when the proportion of ARV-treated PLHIV was lower than 30%, the age of the root node (tMRCA) was reasonably estimated with narrow HPD interval. On the other hand, for the two subclusters (subclusters 6 and 12, Figure 1) with the highest proportions of ARV-treated PLHIV (i.e., 45% and 36%), the 95% HPD intervals for the tMRCA were much wider (95% HPD: 1948.8–1985.3 and 1945.5–1990.2) and the tMRCA was estimated at earlier time periods in 1968.2 and 1971.1 (median estimates) than the remaining 11 subclusters (Figure 1). After the merging of each one of those two subclusters with another subcluster, the tMRCA for the two subclusters of interest were re-estimated at 1990.5 (95% HPD: 1987.2–1993.3) and 1992.6 (95% HPD: 1987.5–1996.4). Notably, molecular clock analysis gave reasonable ancestral time and narrow HPD intervals for the clusters when the proportion of ARV-treated individuals was lower than 30%.

### 3.2. Dating the HIV-1 Epidemic in Greece

Plotting the root-to-tip genetic distance against sampling time revealed significant molecular clock signal in the MTCs with low proportion of ARV-treated PLHIV (i.e., subtype B cluster 21, R = 0.41, *p* ≤ 0.001; cluster 25, R = 0.60, *p* = 0.0068). For MTCs with high proportion of ARV-treated PLHIV, we found no significant signal (i.e., subtype B cluster 24, R = −0.011, *p* = 0.9739; cluster 26, R = 0.18, *p* = 0.4808). After the merging of these MTCs with another MTC that resulted in a decrease in the proportion of ARV-treated individuals, the molecular clock signal was found significant (i.e., subtype B cluster 24, R = 0.50, *p* < 0.001; cluster 26, R = 0.68, *p* < 0.001).

**Figure 2:**
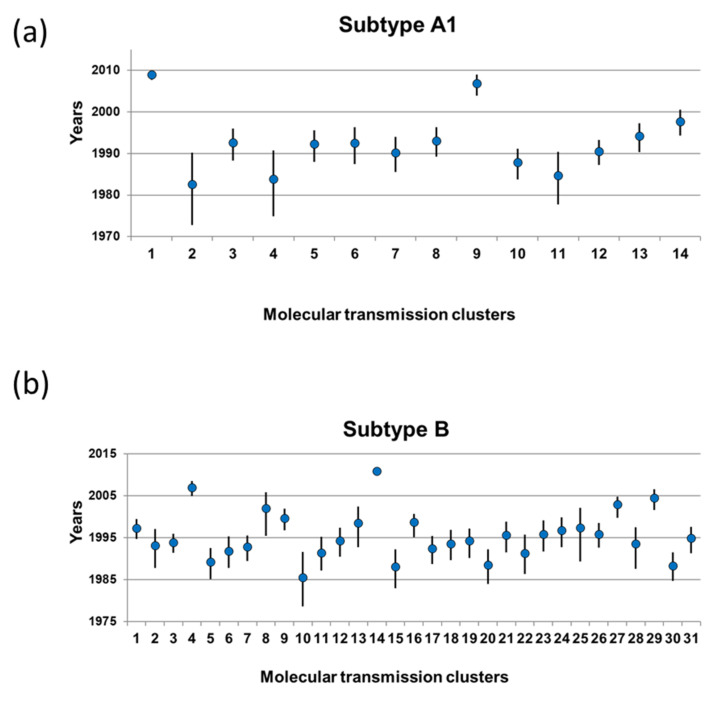
(**a**) Molecular clock calculations of the time of the most recent common ancestor (tMRCA) for the 14 HIV-1 subtype A1 molecular transmission clusters (median, 95% HPD interval). (**b**) Molecular clock calculations of the tMRCA for the 31 HIV-1 subtype B molecular transmission clusters (median, 95% HPD interval). Dots indicate the median estimations and horizontal lines the 95% HPD intervals.

**Figure 3:**
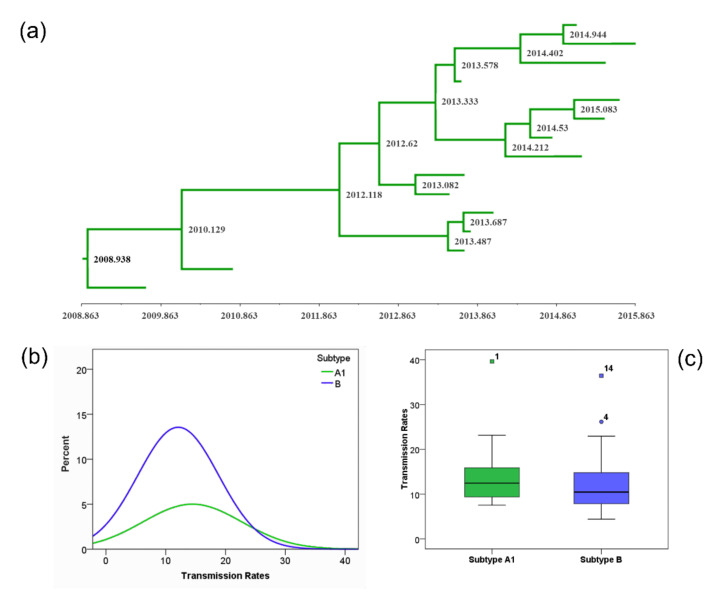
(**a**) Dated tree of the more recent molecular transmission cluster of subtype A1 (cluster 1), estimated by molecular clock analysis; (**b**) Distribution of transmission rates for the subtype A1 and B molecular transmission clusters; (**c**) Boxplots of transmission rates for subtype A1 and B molecular transmission clusters.

We estimated the node ages and the tMRCA at the root node for all the large MTCs using the approach described in detail in the methods section (Figure S1). The characteristics of the PLHIV whose sequences found within those MTCs and analyzed phylodynamicaly, are shown in Table 1. Inclusion of ARV-treated PLHIV was decided to increase the sampling coverage in our study population. The tMRCA of the 14 and 31 subtype A1 and B MTCs, respectively, are shown in Figure 2 and Table 2. The earliest tMRCA dates for subtype A1 were in 1982.6 and for most of the MTCs the estimated dates were between 1987.9 and 1994.2 (IQR). A single MTC was much more recent with the tMRCA to be inferred in 2008.9 (Figure 3a). For subtype B, the earliest tMRCA was in 1985.5, and the time of origin for most of the MTCs was between 1991.8 and 1998.5 (IQR). Similarly to subtype A1, the tMRCA for a single cluster was estimated at 2010.9, suggesting that it was more recent.

### 3.3. HIV Transmission Rates

Given that the majority of sequences within the MTCs have been sampled from Greece, the tMRCA of the tree nodes can be used as a proxy for the HIV infection date using Sanger sequencing data from densely sampled populations. To investigate the rate transmission growth within each MTC, we estimated the transmission rate within each cluster, as described previously [26]. This rate equals the number of infections per the total observation time (person years) for each individual within a cluster since the time of their infection. To avoid potential bias from ARV-treated individuals, transmission rates were estimated only for ARV-treatment naïve PLHIV within the MTCs.

The transmission rate for the 14 MTCs of subtype A1 ranged between 7.54 and 39.61 infections/100 person years (IQR: 9.39, 15.88) (Figure 3b,c, Table 3). For subtype B, transmission rates of the 31 MTCs ranged between 4.42 and 36.44 infections/100 person years (IQR: 7.38, 15.04) (Figure 3b,c, Table 3). Notably, as shown in Table 3, for the MTCs with higher proportion of PWID (i.e., clusters 1 and 9 of subtype A1 and cluster 4 of subtype B), the transmission rate was found to be higher than the rest. To investigate the factors associated with the transmission rate, we performed a multivariable linear regression analysis. Statistical analysis revealed that gender, age, sampling year and risk group were significantly associated with transmission rate. Specifically, an increase in the percentage of males within a MTC and age was associated with a decrease in the transmission rate (gender: coef.: −0.24; 95% CI: −0.43 to −0.05; *p*-value: 0.014; age: coef.: −0.32; 95% CI: −0.60 to −0.04; *p*-value: 0.024), while the most recent sampling was associated with an increase in the transmission rate (coef.: 1.86; 95% CI: 1.31 to 2.41; *p* < 0.001). In addition, MTCs of PWID had a higher transmission rate compared to MTCs of MSM (coef.: 6.73; 95% CI: 0.86 to 12.60; *p* = 0.026). Fast growing MTCs were detected in both central/southern and northern Greece. No differences were observed in transmission rates between the MTCs of subtypes A1 and B. Furthermore, neither the number of sequences per MTC nor the percentage of Greeks within it were associated with transmission rate.

## 4. Discussion

In the current study we found that the local clusters (MTCs) of the most prevalent subtypes that gave rise to HIV-1 infections diagnosed after 1998 in Greece, originated in the early eighties. Specifically, the earliest dates were 1982.6 and 1985.5 for subtypes A1 and B, respectively. These dates correspond to the origin of viral infections without including samples from the early epidemic in the eighties and, therefore, should be considered as the latest boundary of the HIV-1 epidemic origin in Greece. The separation of the largest MTC of subtype A1 in 13 subclusters for the molecular clock estimations (phylodynamic analyses) suggests that the tMRCA of this cluster preceded the time of origin of the individual subclusters. The earliest tMRCA of subtype A1 subclusters that was estimated in 1982.6 was very close to the early epidemic in Greece. This date was in accordance with our previous inference about the time of origin of subtype A1 in Greece in 1978 [27]. Notably, according to surveillance data, HIV epidemic expansion was documented after 1984 in Greece, suggesting that the origin of the locally expanding lineages should be around the early eighties, as estimated in our analysis. Regarding the subtype B, this is the first analysis about the time of the origin of locally expanding clusters in Greece. Although subtype B was the dominant clade circulated at proportions higher than 80% during the eighties in Greece [27], we inferred similar dates to subtype A1.

To the best of our knowledge, our study provides evidence about the earliest subtype A1 lineage circulated in Europe and the Americas dated in early eighties, suggesting that besides subtype B that dominated in Western countries, additional clades have been circulated in Europe. The non-B clades spreading locally in Europe were rare in the eighties [28]. Our previous analysis indicated a lower number of founder strains of subtype A1 versus B; however, the date of introduction of the earliest viruses expanding locally was similar in both subtypes [22]. Subtype A1 in Greece provided a source of infections for other European countries, including Cyprus, Portugal, Spain, France and United Kingdom, where the origin of the main cluster was estimated in 1996 [28]. Another study [29] suggested that a different lineage of subtype A, initially designated as AFSU and currently as A6 sub-subtype, has been introduced from Central Africa to Byelorussia in 1982 [30,31]. The origin of subtype A1 in Greece in the same study was inferred in 1983, which was almost identical to our estimations [27,29]. These findings support that Greece provided the earliest point for nested expansion of subtype A1 in Europe and the Americas.

As regards subtype B, we estimated that the origin of local expansion was in middle eighties. Previous studies, using sequences from earliest available samples, found that the origin of subtype B in the Americas and Europe was in the early and late seventies [32,33,34], respectively. Using a similar sampling frame, the estimated tMRCA of the local clusters in the UK was similar to ours [17].

We found that sequences from ARV-treated individuals can be used in a phylodynamic analysis only if their proportion within the MTC was lower than approximately 30%. ARV-treatment blocks viral replication, causing a pause to viral evolution and thus disturbing the molecular assumption for a roughly constant evolutionary rate over time. We provided evidence that the inclusion of a small proportion of sequences from ARV-treated individuals does not disturb molecular estimations and, on the other hand, provides an advantage for the analysis by improving the sampling (dense sample). Moreover, it has been shown that the molecular clock analysis can be used for the accurate estimation of node ages based on Sanger sequencing data applied for MTCs [35]. Specifically, the molecular clock inferred infection dates were correlated with the clinically estimated ones and there was an agreement between them, suggesting the high accuracy of the molecular clock calculations [35].

Estimation of transmission rates, corresponding to the number of infections per person years within each MTC, provided some interesting features about the epidemic growth of the locally expanding clusters. First, transmission rates were significantly higher for MTCs consisting of younger PLHIV, for MTCs consisting of PWID and for MTCs including sequences obtained more recently. Greece experienced a large outbreak among PWID ignited in late 2010 and PWID clusters were sampled during that period, therefore suggesting that the higher transmission rates were due to the increased incidence rate among this group during the outbreak period [23]. We would expect that heterosexuals would be found more frequently among slowly expanding clusters; however, in some cases the risk group had been misreported due to stigma (unpublished data), therefore making difficult to investigate the actual effect of this risk group to transmission rate. Higher transmission rates were detected also for PLHIV with more recently sampled sequences that could be due to more risky behavior in recent years. This hypothesis cannot be confirmed since behavior data are not available in our study. In addition, no differences were detected between subtypes A1 and B, suggesting that the increasing trend of subtype A1 versus B during the previous decade [27] was probably not due to a higher transmission efficiency of subtype A1. As we previously reported, no significant differences were found in the proportion of risk groups or any other populations’ characteristic between subtypes A1 and B. Given the similar characteristics of the two populations [22], the most plausible hypotheses for the difference in trend of the two subtypes were that either subtype A1 was more infectious or that this difference was due to a higher risk behavior of those infected with subtype A1. Our results that transmission rates did not differ between the two subtypes support the hypothesis that in our setting subtype A1 was probably not more infectious than B. Therefore, the most plausible hypothesis for the increasing trend of subtype A1 was probably due to the higher risk behavior of the PLHIV and their partners infected with this subtype. This was further supported by the finding that the highest transmission rate was observed within PWID clusters during the outbreak, when local conditions favored high transmission rates among this highly vulnerable group [23,36]. Additional data about risky behavior are needed to confirm the hypothesis about the reasons for the increasing trend of subtype A1.

Our study has some limitations, among which is that HIV-1 sequences were sampled after 1998, suggesting that the estimated tMRCA should be considered as the latest boundary of the epidemic origin of subtypes A1 and B in Greece. Moreover, data about the study participants’ risky behavior are missing. The strengths of our study include the dense sampling, which accounts for 57.2% of all people diagnosed with HIV-1 during the same time period in Greece, and the multiple validation steps of the molecular clock analysis, including the analysis about the effect of the ARV-treated population and the validation of the inferred infection dates using sequences from PLHIV with previously known infection dates from a different source, as described previously [35].

In conclusion, we estimated that the latest dates of the locally expanding subtype A1 and B lineages in Greece were in the beginning of eighties, providing evidence about the earliest subtype A1 epidemic in Europe. Transmission rates were significantly higher for PWID clusters due to the conditions that gave rise to an extensive PWID HIV outbreak ten years ago in Athens, Greece. Transmission rate can be considered a valuable measure for public health since, in addition to other parameters, it provides a proxy of the rate of epidemic growth within a cluster and, therefore, it can be useful for targeted HIV prevention programs. Our study provides a nationwide study about the dating of the HIV epidemic as well as some important characteristics of the HIV-1 epidemic transmission in Greece, that in addition to newly accumulated data can be used for targeted HIV-1 prevention in the future.

## Figures and Tables

**Table 1:** Demographic characteristics of a subset of the study population (i.e., PLHIV infected within major molecular transmission clusters).

Characteristic	
Median age [in years, (IQR)]	35.2 (29.2–43.3)
HIV subtype, *n* (%)	
A1	1533 (49.6)
B	1560 (50.4)
Gender, *n* (%)	
Male	2708 (87.5)
Female	272 (8.8)
Unknown	113 (3.7)
Transmission risk group, *n* (%)	
MSM ^1^	2110 (68.2)
PWID ^2^	236 (7.6)
Heterosexuals	505 (16.3)
Other	23 (0.8)
Unknown	219 (7.1)
Antiretroviral therapy, *n* (%)	
Naive	2429 (78.5)
Treated	501 (16.2)
Unknown	163 (5.3)
Ethnicity, *n* (%)	
Greek	2527 (81.7)
Non-Greek	211 (6.8)
Unknown	355 (11.5)
Region of sampling, *n* (%)	
Northern Greece	694 (22.4)
Central/Southern Greece	2351 (76.0)
Crete	48 (1.6)
Total, *n* (%)	3093 (100.0)

^1^ MSM: men who have sex with men; ^2^ PWID: people who inject drugs.

**Table 2:** Time of the most recent common ancestor (tMRCA) in years of subtype A1 and B molecular transmission clusters.

**Subtype A1 cluster number**	**tMRCA, years, median (95% HPD ^1^)**
1	2008.94 (2007.70–2009.68)
2	1982.58 (1972.80–1990.21)
3	1992.65 (1988.29–1996.01)
4	1983.84 (1974.87–1990.76)
5	1992.35 (1988.02–1995.56)
6	1992.56 (1987.46–1996.37)
7	1990.17 (1985.50–1994.03)
8	1993.07 (1989.22–1996.28)
9	2006.84 (2003.93–2008.95)
10	1987.85 (1983.71–1991.18)
11	1984.71 (1977.77–1990.40)
12	1990.47 (1987.23–1993.29)
13	1994.18 (1990.28–1997.25)
14	1997.70 (1994.30–2000.50)
**Subtype B cluster number**	**tMRCA, years, median (95% HPD)**
1	1997.28 (1994.73–1999.41)
2	1993.13 (1987.73–1997.03)
3	1993.86 (1991.37–1995.89)
4	2006.96 (2004.92–2008.55)
5	1989.18 (1985.08–1992.52)
6	1991.81 (1987.76–1995.30)
7	1992.83 (1989.42–1995.53)
8	2002.06 (1995.41–2005.87)
9	1999.64 (1996.72–2001.89)
10	1985.47 (1978.57–1991.58)
11	1991.43 (1987.12–1995.18)
12	1994.31 (1990.45–1997.40)
13	1998.52 (1992.76–2002.46)
14	2010.90 (2009.94–2011.43)
15	1988.09 (1982.96–1992.19)
16	1998.71 (1995.06–2000.70)
17	1992.40 (1988.70–1995.47)
18	1993.56 (1989.62–1996.87)
19	1994.30 (1990.20–1997.13)
20	1988.47 (1983.94–1992.19)
21	1995.63 (1991.51–1998.85)
22	1991.33 (1986.36–1995.70)
23	1995.86 (1991.72–1999.11)
24	1996.77 (1992.69–1999.85)
25	1997.34 (1989.30–2002.14)
26	1995.87 (1992.66–1998.53)
27	2002.95 (1999.74–2004.81)
28	1993.55 (1987.53–1997.52)
29	2004.48 (2001.66–2006.58)
30	1988.32 (1984.63–1991.53)
31	1994.86 (1991.27–1997.55)

^1^ HPD: highest posterior density.

**Table 3:** Transmission rates and characteristics of the different molecular transmission clusters in Greece.

Cluster Number	Number of Sequences	HIV-1 Subtype	Transmission Rate (Number of Transmissions/100 Person Years)	Region of Sampling (Athens, %)	Age (Median)	Gender (Male, %)	Risk Group (%)		Ethnicity (Greek, %)
1	15	A1	39.608	0.0	35.5	53.3	MSM	6.7	46.7
							PWID	33.3	
							HETERO	6.7	
2	68	A1	9.390	61.2	34.0	76.5	MSM	58.8	75.0
							PWID	2.9	
							HETERO	23.5	
3	124	A1	13.702	90.3	36.0	95.2	MSM	76.6	90.3
							PWID	1.6	
							HETERO	17.4	
4	83	A1	8.736	84.3	37.0	96.4	MSM	89.2	94.0
							PWID	1.2	
							HETERO	6.0	
5	83	A1	12.870	83.1	36.5	77.1	MSM	62.7	85.5
							PWID	3.6	
							HETERO	30.1	
6	28	A1	7.540	75.0	40.5	67.9	MSM	53.6	71.4
							PWID	0.0	
							HETERO	35.7	
7	164	A1	11.498	34.4	38.0	84.2	MSM	67.1	76.2
							PWID	0.6	
							HETERO	17.7	
8	93	A1	12.043	83.9	49.0	81.7	MSM	52.7	78.5
							PWID	4.3	
							HETERO	33.3	
9	66	A1	23.146	95.5	35.0	87.9	MSM	0.0	83.3
							PWID	95.5	
							HETERO	1.5	
10	348	A1	13.068	57.2	37.0	84.5	MSM	65.2	80.5
							PWID	3.7	
							HETERO	21.6	
11	120	A1	8.586	50.9	38.0	79.2	MSM	58.3	72.5
							PWID	5.0	
							HETERO	25.0	
12	74	A1	10.406	93.2	44.0	78.4	MSM	50.0	60.8
							PWID	1.4	
							HETERO	36.5	
13	208	A1	15.880	84.6	31.0	93.8	MSM	85.1	82.2
							PWID	0.5	
							HETERO	7.7	
14	59	A1	15.911	88.1	30.0	98.3	MSM	89.8	91.5
							PWID	3.4	
							HETERO	5.1	
1	180	B	16.519	87.2	31.0	98.3	MSM	88.9	84.4
							PWID	0.0	
							HETERO	4.4	
2	34	B	9.447	85.3	33.0	94.1	MSM	82.4	91.2
							PWID	0.0	
							HETERO	14.7	
3	54	B	15.038	98.2	35.0	72.2	MSM	57.4	87.0
							PWID	3.7	
							HETERO	35.2	
4	112	B	26.152	99.1	34.0	83.9	MSM	0.9	80.4
							PWID	98.2	
							HETERO	0.9	
5	43	B	6.439	79.1	37.0	53.5	MSM	32.6	88.4
							PWID	4.7	
							HETERO	55.8	
6	13	B	6.295	100.0	37.0	92.3	MSM	76.9	84.6
							PWID	0.0	
							HETERO	23.1	
7	17	B	6.207	76.5	42.5	76.5	MSM	64.7	64.7
							PWID	5.9	
							HETERO	17.7	
8	11	B	9.335	100.0	34.0	100.0	MSM	100.0	72.7
							PWID	0.0	
							HETERO	0.0	
9	11	B	7.376	27.3	55.0	54.6	MSM	36.4	81.8
							PWID	0.0	
							HETERO	54.6	
10	76	B	10.452	37.3	40.0	84.2	MSM	54.0	75.0
							PWID	0.0	
							HETERO	25.0	
11	30	B	11.373	83.3	37.0	96.7	MSM	96.7	83.3
							PWID	0.0	
							HETERO	3.3	
12	11	B	4.469	63.6	39.5	90.9	MSM	72.7	90.9
							PWID	0.0	
							HETERO	27.3	
13	31	B	14.551	96.8	30.5	100.0	MSM	90.3	87.1
							PWID	0.0	
							HETERO	6.5	
14	25	B	36.436	92.0	32.0	96.0	MSM	88.0	76.0
							PWID	0.0	
							HETERO	0.0	
15	53	B	8.334	13.2	35.0	83.0	MSM	64.2	66.0
							PWID	5.7	
							HETERO	15.1	
16	39	B	16.677	94.9	33.0	100.0	MSM	94.9	97.4
							PWID	0.0	
							HETERO	5.1	
17	56	B	10.424	75.0	38.5	89.3	MSM	66.1	80.4
							PWID	5.4	
							HETERO	17.9	
18	12	B	4.415	91.7	39.5	91.7	MSM	66.7	100.0
							PWID	8.3	
							HETERO	25.0	
19	28	B	9.219	100.0	32.5	78.6	MSM	60.7	75.0
							PWID	7.1	
							HETERO	28.6	
20	137	B	11.978	92.7	34.0	92.0	MSM	79.6	86.9
							PWID	0.7	
							HETERO	16.1	
21	122	B	13.722	76.2	32.0	89.3	MSM	77.1	86.1
							PWID	0.0	
							HETERO	10.7	
22	66	B	16.164	89.2	30.0	95.5	MSM	86.4	84.9
							PWID	1.5	
							HETERO	4.6	
23	20	B	8.546	85.0	46.0	80.0	MSM	70.0	80.0
							PWID	0.0	
							HETERO	25.0	
24	12	B	5.847	83.3	35.0	100.0	MSM	91.7	66.7
							PWID	0.0	
							HETERO	8.3	
25	19	B	9.814	89.5	40.0	100.0	MSM	84.2	94.7
							PWID	0.0	
							HETERO	10.5	
26	18	B	6.480	94.4	35.0	83.3	MSM	66.7	83.3
							PWID	5.6	
							HETERO	16.7	
27	9	B	15.993	88.9	45.5	88.9	MSM	77.8	66.7
							PWID	0.0	
							HETERO	22.2	
28	25	B	11.309	84.0	33.5	92.0	MSM	68.0	60.0
							PWID	4.0	
							HETERO	8.0	
29	31	B	22.943	54.8	36.0	93.6	MSM	77.4	71.0
							PWID	0.0	
							HETERO	0.0	
30	218	B	10.864	90.4	34.0	94.0	MSM	83.0	87.2
							PWID	1.4	
							HETERO	12.8	
31	47	B	11.874	87.2	38.0	83.0	MSM	78.7	93.6
							PWID	2.1	
							HETERO	17.0	

## Data Availability

The data presented in this study are available on request from the corresponding author. The whole data set corresponds to a dense sampling of PLHIV in Greece. Therefore, the data are not publicly available to avoid the risk of PLHIV identification.

## Supplementary Materials

The following are available online at https://www.mdpi.com/article/10.3390/v14010101/s1. Figure S1: Dated phylogenetic trees of HIV-1 sequences found within A. Subtype A1 and B. Subtype B molecular transmission clusters (MTCs), estimated by molecular clock analysis implemented in BEAST program (version 1.8.0).
