# Supplementary material for: Dating the Origin and Estimating the Transmission Rates of the Major HIV-1 Clusters in Greece: Evidence about the Earliest Subtype A1 Epidemic in Europe
Source: Viruses. 2022 Jan 6;14(1):101. doi: 10.3390/v14010101 (PMC8782043; doi:10.3390/v14010101)
Supplement: Supplementary file 1 [file viruses-14-00101-s001.zip › viruses-1460295-supplementary.pdf]

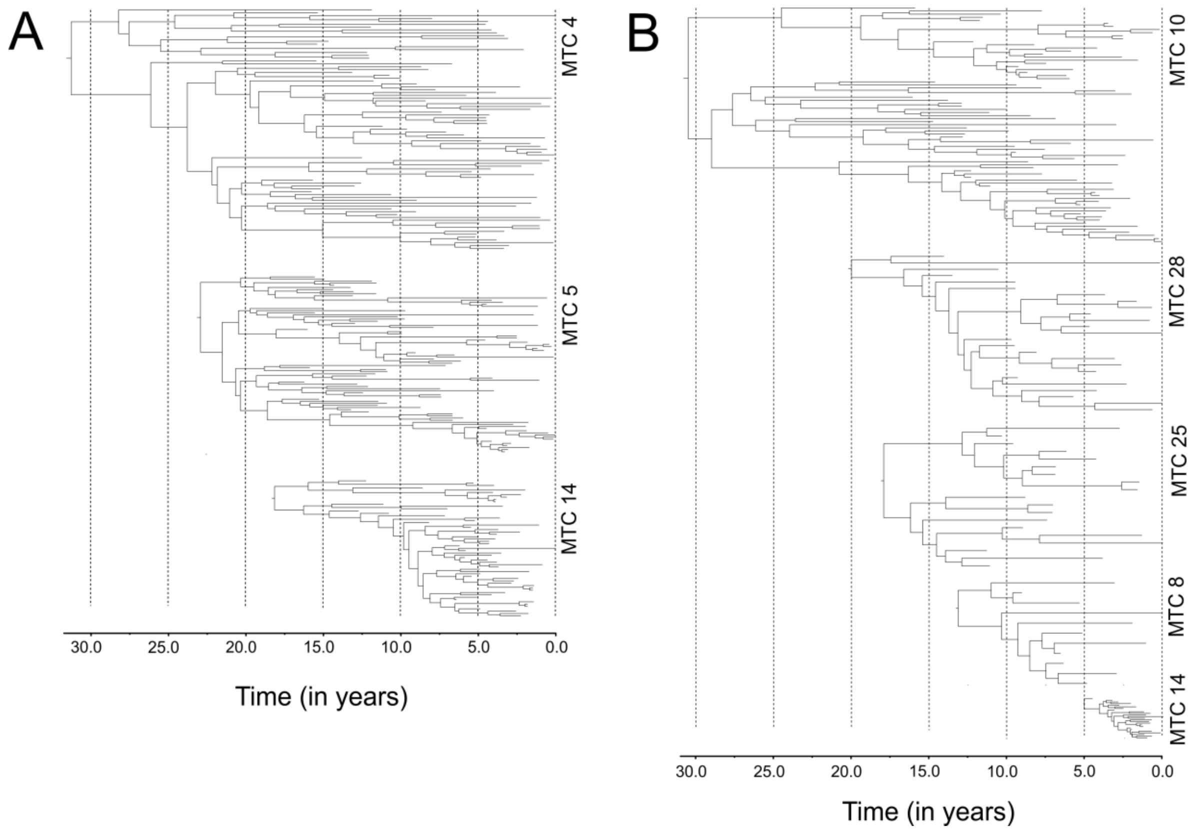

Figure S1: Dated phylogenetic trees of HIV-1 sequences found within A. Subtype A1 and B. Subtype B molecular transmission clusters (MTCs), estimated by molecular clock analysis implemented in BEAST program (version 1.8.0).
